# Eating lizards: a millenary habit evidenced by Paleoparasitology

**DOI:** 10.1186/1756-0500-5-586

**Published:** 2012-10-25

**Authors:** Luciana Sianto, Isabel Teixeira-Santos, Marcia Chame, Sergio M Chaves, Sheila M Souza, Luiz Fernando Ferreira, Karl Reinhard, Adauto Araujo

**Affiliations:** 1 Fundação Oswaldo Cruz, Avenida Brasil 4365, Manguinhos, CEP 21040-900, Rio de Janeiro, RJ, Brasil; 2 University of Nebraska-Lincoln School of Natural Resources, 719 Hardin Hall, 3100 Holdrege Street, Lincoln, NE 68583-0987, USA

**Keywords:** Paleoparasitology, Coprolite, Helminths, Lizard, Zoonosis

## Abstract

**Background:**

Analyses of coprolites have contributed to the knowledge of diet as well as infectious diseases in ancient populations. Results of paleoparasitological studies showed that prehistoric groups were exposed to spurious and zoonotic parasites, especially food-related. Here we report the findings of a paleoparasitological study carried out in remote regions of Brazil’s Northeast.

**Findings:**

Eggs of Pharyngodonidae (Nematoda, Oxyuroidea), a family of parasites of lizards and amphibians, were found in four human coprolites collected from three archaeological sites. In one of these, lizard scales were also found.

**Conclusions:**

Through the finding of eggs of Pharyngodonidae in human coprolites and reptile

scales in one of these, we have provided evidence that humans have consumed reptiles at least 10,000 years ago. This food habit persists to modern times in remote regions of Brazil’s Northeast. Although Pharyngodonidae species are not known to infect humans, the consumption of raw or undercooked meat from lizards and other reptiles may have led to transmission of a wide range of zoonotic agents to humans in the past.

## Findings

As in modern times, diet varied among prehistoric human groups and small animals, including lizards and other reptiles, were important food sources for prehistoric people [1,2]. Analyses of human coprolites demonstrate this dietary diversity. Coprolites also contain intestinal parasites transmitted by contaminated food or water, including zoonotic helminths [3]. Moreover, spurious parasites can also be found in human coprolites and continue to occur, especially in current groups with traditional food habits [4]. By spurious, we mean parasite eggs that are not infective to humans and pass harmlessly through the human intestinal tract. *Parapharyngodon sceleratus* Chatterji, 1933 (Oxyuroidea: Pharyngodonidae) eggs were recorded in lizard coprolites (*Tropidurus torquatus*, Squamata, Tropiduridae) dating from 9,000 to 11,000 years [5]. The diagnosis was based on egg morphology and metric parameters in comparison with published checklists. The parasite is known to commonly occur in several lizard and amphibian species of the American continent [6-10].

**Figure 1:**
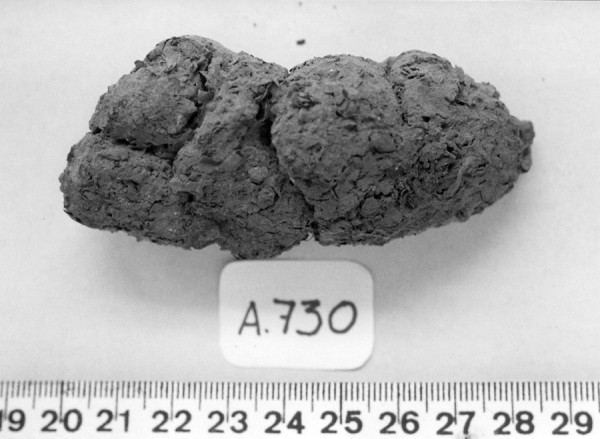
Human coprolite found in burial dated between 1860 ± 50 and 1610 ± 70 years BP from archaeological site of Furna do Estrago, northeastern Brazil.

Herein we describe the finding of Pharyngodonidae eggs in four coprolites, morphologically identified as specimens of human origin (Figure 1) according to Chame [11]. These coprolites were collected during excavations in the Archaeological Area of São Raimundo Nonato (n=3) and in the archaeological site of Furna do Estrago (n=1), situated in the states of Piauí and Pernambuco, respectively. Piauí and Pernambuco are neighboring states of Brazil located in the northeastern part of the country [12]. The dates of coprolites were based on radiocarbon technique applied to bones from layers and burials where coprolites were found. The method adopted in paleoparasitological analysis was applied to each sample as follows. Five grams from each coprolite were removed and placed in receptacles with 0.5% aqueous trisodium phosphate solution for 72 hours for rehydration [13]. After this period, the fragments were homogenized with a glass rod and strained through a funnel with triple-folded gauze into conical glass jars [14]. The content retained in gauze was analyzed for food remains with a stereomicroscope. After 24 h, twenty slides were prepared from the sediment in the bottom of each jar for optical microscope analysis. Slides were observed at 100x and 400x magnifications. 

The first coprolite was collected in the archaeological layer dated to 10,640 ± 80 years before present (BP) and located in the site known as Toca dos Coqueiros (Archaeological Area of São Raimundo Nonato). Two other samples were collected in the site of Toca da Baixa dos Caboclos (Archaeological Area of São Raimundo Nonato) from two human burials dated respectively 525 to 315 (Beta 136209) and 530 to 440 (Beta 136208) cal years BP.

A fourth coprolite was collected in the archaeological site of Furna do Estrago (Pernambuco state), which is located in an area of upland forests, a mesic enclave, representing a true "oasis" in a semiarid region. The coprolite was found in a human burial dated between 1860 ± 50 (Beta 145954) and 1610 ± 70 (Beta 145955) years BP [15].

**Figure 2:**
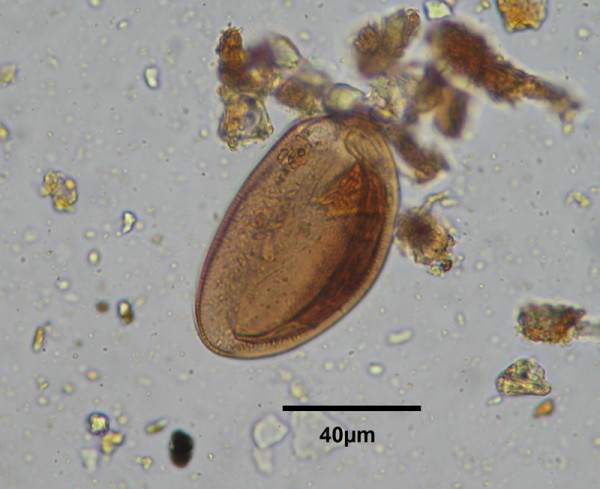
Pharyngodonidae eggs in human coprolites from northeastern Brazil.

In all four coprolites, nematode eggs were found, two eggs in each coprolite from Toca da Baixa dos Caboclos and one egg into each other, measuring 76.9 (SD 8.1) × 43 (SD 6.6) μm (n=6). First, eggs were identified as oxyurid eggs. Then, morphological characters of eggs found in human coprolites (Figure 2) were compared with those of oxyurid eggs available in literature [16-18]. Eggs found in coprolites presented a striated brown shell formed in three layers and size range between 62,5-85,7 × 35–51, 2 μm, similar to those of oxyurid eggs from reptiles [6,19]. Since these eggs were similar in shape and size to those formerly recorded in lizards [20], including in lizard coprolites [5,21], they were identified as eggs of Pharyngodonidae, likely of Parapharyngodon sp. or Pharyngodon sp., both genera of intestinal parasites of reptiles and amphibians. A more accurate diagnosis was not possible because of the similarities between the eggs of these two genera [22] that parasitize several lizard species in Brazil [7,23]. 

A careful analysis of food remains was carried out. All coprolites showed macroscopic and microscopic remains consistent with those expected for a typical human diet, such as seeds, plant fibers, cooked starch grains, phytoliths, charcoal remains, and pollen grains. These latter were identified as belonging to the families Chenopodiaceae, Malvaceae and Convolvulaceae. Some species in these families are known for their medicinal properties and have traditionally been used, even up to the present day, to treat inflammation, abscesses, intestinal disorders, and parasites [24,25].

**Figure 3:**
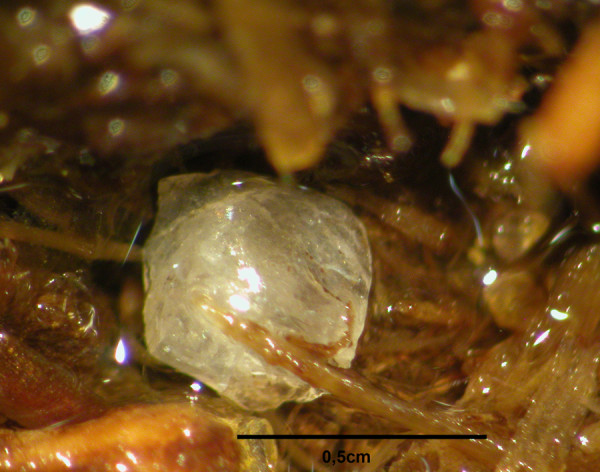
Lizard scale found in human coprolite from burial dated 525 to 315 cal years BP, from archaeological site of Toca da Baixa dos Caboclos, northeastern Brazil.

In one of the two human burial coprolites from Toca da Baixa dos Caboclos, small iridescent plates, identified as reptile scales, were also found (Figure 3). The scales didn’t present evidence of burn or cooking. First, they were identified as reptile scales. Then scales were compared microscopically in our lab with scales belonging to our reference collection. Our zoological reference collections include a lizard found dead in same area during a field expedition. Based on the morphological similarities, scales were securely identified as lizard. Based on the finding of eggs of typical reptile parasites (without known zoonotic potential) together with lizard scales in the same coprolite, we concluded that an individual had ingested one or more lizard hosts of Pharyngodonidae adult parasites. This caused Pharyngodonidae eggs to be released in the human’s intestinal tract. The undigested eggs were spuriously present this ancient human. Thus, lizard remains and Pharyngodonidae eggs were associated in this human burial coprolite.

Hunter-gatherers groups in different parts of the world consume freshly killed animals or living animals for their subsistence, including reptiles. The biological risks associated with consumption of reptile meat has been reviewed by Magnino et al. [26] and includes diseases caused by bacteria, viruses, intestinal helminths and other parasites. Forty-six species of lizards are reported in the Brazilian Caatinga, including large species such as *Iguana iguana* and *Tupinambis merianae* and many others of medium and small size [27,28]. They represent an important biomass and a food source available for human groups. The consumption of lizards is a relatively common practice in parts of Brazilian northeast semi-arid region, especially during periods of prolonged drought, when the population is forced to search alternative food resources [29]. Today, in this study area, children and young people capture small lizards for complementary feeding of families. Anthropologically, this suggests that prehistoric people in the semiarid region of Brazil used small animals as food source similarly to hunter-gatherers and agriculturalists in North America, as discussed by Reinhard et al. [1] and Sutton and Reinhard [30]. It is noteworthy that in some areas of Brazil, this strategy is still used currently.

Even arthropods potentially able to transmit parasitic disease, such as ticks and fleas, were reported in human coprolites [31]. Humans can also ingest head lice in the process of grooming hair [32].

Results of the present analysis of human coprolites bring further data about spurious parasitism and food habits in prehistoric populations. We found eggs of reptilian parasites and lizard remains in human coprolites dating from 10,000 years ago to colonial times. This shows that the habit of eating lizards, still persistent in the current local population, had ancient origins in the Brazilian semiarid region. Moreover, although species of parasites belonging to the Pharyngodonidae family are not known to infect humans, it is possible that the consumption of lizard meat led to the risk of human infection by a wide variety of zoonotic agents in the past [26].

## Competing interests

The authors declare that they have no competing interests.

## Authors' contributions

LS, MC, AA and LFF performed paleoparasitological analysis and parasite identifications. LS, AA, ITS prepared the first versions of the manuscript, and LFF and KR reviewed the final versions. Diet remains were analysed by ITS and KR. SMS helped with archaeological contextualization. All authors participated in obtainment of financial support for research and drafting of the manuscript. All authors have read and approved the final manuscript.
